# Improvement in Fatigue, Sleepiness, and Health-Related Quality of Life with Bright Light Treatment in Persons with Seasonal Affective Disorder and Subsyndromal SAD

**DOI:** 10.1155/2011/543906

**Published:** 2011-06-13

**Authors:** Cecilia Rastad, Jan Ulfberg, Per Lindberg

**Affiliations:** ^1^Department of Neuroscience, Uppsala University, 75124 Uppsala, Sweden; ^2^Center for Clinical Research Dalarna (CKF), Nissers väg 3, 791 82 Falun, Sweden; ^3^Sleep Lab, Inland Hospital, 2500 Tynset, Norway; ^4^Department of Psychology, Uppsala University, 752 37 Uppsala, Sweden

## Abstract

*Objective*. To investigate the effects of bright light treatment for secondary outcome measures and to explore and validate empirically derived subgroups and treatment effects in subgroups. *Methods*. A descriptive design. A sample of forty-nine persons (mean age of 45.8) with clinically assessed seasonal affective disorder (SAD) or subsyndromal SAD (S-SAD) participated in a two-group clinical trial evaluating the effects of treatment with bright light therapy. A person-oriented cluster analysis was applied to study treatment effects in subgroups. *Results*. For the merged group, sleepiness (Epworth Sleepiness Scale), fatigue (fatigue questionnaire), and health-related quality of life (SF-36) were improved at posttreatment, and results were maintained at the one-month followup. Three distinct subgroups had a high level of fatigue in common, while the level of excessive daytime sleepiness and depressed mood differed between the subgroups. Over time, all subgroups improved following ten days treatment in a light room. *Conclusion*. Fatigue, excessive daytime sleepiness, and health-related quality of life improve in a similar way as depressed mood following treatment with bright light. The treatment was effective irrespective of the severity of the disorder, that is, for persons with SAD and subsyndromal SAD.

## 1. Introduction

Seasonal variation in mood and behaviour (seasonality) is common [1, 2] and is associated with self-reports of anxiety and depression [3]. It has been suggested that a combination of vulnerability to both seasonality and depression may be related to seasonal affective disorder (SAD), a seasonal subtype of major depression [4, 5]. Different combinations of vulnerability for the two presentations may explain the variety of symptoms found in SAD and in the milder form of SAD, subsyndromal SAD (S-SAD) [6]. The recurring seasonal symptoms are triggered by changes in environmental factors, especially reduced daylight in winter [7]. Replacing some of the deficient daylight during the winter season is one way of managing the symptoms. Treatment with strong artificial light, bright light therapy (BLT), has shown promising results in SAD [8, 9]. There is some evidence that BLT is effective in S-SAD as well [10].

The majority of studies investigating the effects of BLT have involved the use of light boxes, that is, portable devices that can be used for treatment in patients' home environment. Another form of BLT is provided in light rooms, originally designed for research purposes at the Karolinska Institute in Stockholm (Sweden) in order to obtain accurate measurements of the light dose in different experiments concerned with the hormonal effects of light in humans [11]. This concept was further evolved into light rooms for treatment studies in Sweden in the 1980s and 1990s in order to improve the control of treatment compliance, to be able to treat a group of patients (and not one individual at a time) and to be able to follow the symptoms of each patient on a daily basis (L. Wetterberg, pers. comm. 2009-01-11). Light rooms for research purposes were developed in Finland during the same time period [12]. When using a light box, the light intensity is strongly influenced by the distance between the eyes and the light source [13, 14]. In a light room, the light is indirect and evenly distributed in the room, and therefore it is possible to obtain more reliable estimates of the light dose (duration × intensity) compared to when the light box is used. During treatment, which is carried out in a similar way as a treatment with the light box (i.e., daily treatments in the morning for ten consecutive days including followup treatments when appropriate), patients sit comfortably on chairs reading, writing, or talking. There is no standard for the light intensity in a light room, and consequently, these vary approximately between 1500 and 6000 Lux (clinical experience). Because of the differences between these two modes of treatments, results from clinical studies using light boxes cannot automatically be applied to light room treatments. These two forms of BLT certainly have the bright light in common but differ in several other aspects that may affect outcome. For example, staff and other patients are present in the light room, while family members may be present during treatment in home environments. The majority of light rooms in Swedish health care are situated in psychiatric clinics, a few in primary health care or at sleep disorders clinics. An estimate showed that 39 out of 99 responding psychiatric departments provided light room treatments, which indicate that it is a fairly common treatment in Sweden [15]. When the Swedish Council on Technology Assessments in Health Care (the SBU) reviewed the evidence for BLT in 2004 and 2007, they concluded that the evidence for BLT in general and treatment in a light room in particular was not sufficient and called for additional controlled clinical trials [15, 16]. Up to that date, there were only two publications of clinical trials concerned with treatment in light rooms, one in 1993 by Partonen et al. in Finland [12] and the other in 1995 by Thalén et al. in Sweden [17].

More recently, a randomized controlled clinical trial including a waiting-list control condition showed that BLT (treatments given in four light rooms) had a positive effect on depressive mood in persons with SAD and S-SAD and that results were maintained during the one-month followup [18]. Similar to most clinical studies concerned with the treatment of SAD and S-SAD, the outcome measure in that study was depressive mood. However, since the symptoms in S-SAD may present as tiredness, fatigue, and sleep problems rather than depressed mood [1], it may be of importance to assess other variables when evaluating the effects of the intervention [19]. Therefore, in the above clinical study, reliable and valid measures of excessive daytime sleepiness, fatigue, and health-related quality of life (HRQOL) were used in addition to measures of depressed mood in order to evaluate treatment effects of bright light in a more comprehensive way.

Cluster analysis is a multivariate statistics method that has been used in psychiatric and other research in order to discover “natural subgroups” and to create empirically based classification of clinical syndromes [20]. Would such an analysis, performed in the present sample with SAD and S-SAD and based on a broader range of measures, result in subgroups that were clinically meaningful and useful? Results from an early study of seasonality in the population using cluster analysis and published by Bartko and Kasper in 1989 identified eight clusters/subgroups among persons with seasonal changes in mood and behaviour [21]. The cluster analysis in that study was based solely on results for the Seasonal Pattern Assessment Questionnaire (SPAQ) [6], and two of the clusters were identified as winter SAD and S-SAD. 

In the present study, the aim was to investigate the use of more elaborated outcome measures in clinical trials of BLT, and at the same time to explore and empirically validate subgroups in that sample with SAD and S-SAD. The study was performed in several interrelated steps. More specific research questions were to (i) study the effects of bright light therapy on daytime sleepiness, fatigue, and health-related quality of life, (ii) identify and explore subgroups with cluster analysis based on measures of depression, daytime sleepiness, and fatigue, and (iii) validate these subgroups on cluster variables, other independent variables, and over time.

## 2. Method

### 2.1. Subjects and Procedure

Fifty subjects with SAD or S-SAD, forty women and ten men (mean age of 45.8, range of 20–68 years) living in the Swedish county of Dalarna (lat 60.5), were recruited from a previous population-based prevalence study [2]. In that study, subjects (*N* = 312) scoring above the commonly used cutoffs for SAD and S-SAD on the Seasonal Pattern Assessment Questionnaire (SPAQ) [22] were interviewed by telephone (first author) and then clinically assessed by a psychiatrist in a face-to-face interview (*N* = 91). Subjects were included if they fulfilled criteria for SAD, that is, a major depressive disorder with a winter seasonal pattern (DSM-IV) [4] or S-SAD according to the commonly used Kasper criteria [22]. Exclusion criteria were severe psychiatric or somatic disorder, prescribed antidepressive medication, an eye condition that could be negatively affected by bright light, pregnancy, shift work, previous treatment with BLT, or inadequate knowledge of the Swedish language. 

When subjects experienced relapse of winter depressive symptoms, baseline data were collected with subsequent randomisation to either ten days BLT or a 3-week waiting-list control condition followed by BLT. All data were collected the week before treatment, the week after treatment ended, and at the one-month followup. The study was conducted during two subsequent winter seasons. Results for the main outcome variable (depressive mood) and details of demographic variables, subjects' progression throughout the study, the intervention, and attrition are reported elsewhere [18]. 

In the present study, baseline data for the total group (*N* = 49) were used in the cluster analysis. Complete data (i.e., baseline, posttreatment, and followup) were available for 47 out of 49 subjects; one subject declined participation after baseline, and one subject was lost to posttreatment data due to a short period of somatic ill health. Written informed consent was obtained from all participants prior to their inclusion in the study. The study was approved by the Research Ethics Committee of the Faculty of Medicine at Uppsala University, Sweden.

### 2.2. Measures

The Structured Interview Guide for the *Hamilton Depression Rating Scale-Seasonal Affective Disorders self-rating version* (SIGH-SAD/SR) [23] is one of the most commonly used outcome measures in clinical trials of BLT. It consists of two subscales, the 21-item HAM-D (HAMD-21) and the 8-item atypical symptom score (Atyp-8). Since the two subscales measure different aspects of SAD, general depressive symptoms, and symptoms specific for SAD, the scores of each subscale were used. 

The *fatigue questionnaire* (FQ) was originally developed within a primary care setting [24]. Eleven items concern fatigue experienced during the last month (was changed to the last week in the present study), and two items concern the duration of fatigue. The total score varies from 0 to 33, and it was this score that was used for the analysis. Age- and gender-specific norms for the general population have been published [25].

The *Epworth Sleepiness Scale* (ESS) is a self-report questionnaire commonly used in sleep research as a measure of persistent day-time sleepiness [26]. The eight items cover active and passive situations in which the risk of falling asleep or “dozing off” is estimated on a scale ranging from 0 to 3. The sum scores range from 0 to 24. 

The *SF-36* is a measure of health-related quality of life (HRQOL) in the population, but is used as an outcome measure in treatment studies as well [27]. The thirty-six items are summed up in 8 subscales; the first four subscales constitute the physical component summary scale (PCS) and the last four subscales the mental component summary scale (MCS). The seven-day version of the SF-36 was used in the present study. Norms for the general Swedish population and different subgroups are available [27]. 

Two bivariate *visual analogue scales* (VASs) measuring mood (very sad-very happy) and sleepiness (very sleepy-very alert) were used. Subjects were instructed to put a mark according to the present situation on a 100 mm vertical line with end-point values only. The VAS scales were filled out daily during one week at baseline, posttreatment, and followup. The mean weekly value for each individual was used in the analysis. 

The *Seasonal Pattern Assessment Questionnaire* (SPAQ) was developed during the 1980s as a screening instrument for seasonal symptoms [6]. The part most commonly used in the SPAQ is the global seasonal score (GS-score), a sum score of 6 items concerned with seasonal changes in energy level, mood, sleep length, social activity, weight, and appetite. These items are scored from 0 to 4 (no change-extremely marked change) and the total GS score varies from 0 to 24.

### 2.3. Relations between Measures

Relations between measures were analyzed with Pearson correlation coefficients. If several outcome measures were to be used in the cluster analysis, the correlations between them would have to be moderate. Correlations <0.7 are preferred since they indicate that measures are related but not identical [28]. Even though the correlations were within the preferred range, the ESS had a surprisingly low correlation to the other measures (Pearson *r *(range) = 0.0–0.2).

### 2.4. Statistical Analysis

The few single items missing (<0.5%) in the SIGH-SAD/SR were imputed using the method of last value carried forward [29] or with zero if missing at baseline. The few missing single items (<0.5%) in the SF-36 were imputed according to the manual [27]. For VAS, a minimum of 5/7 daily scorings were considered sufficient. There were no single items missing in the VAS, ESS, or FQ. Treatment effects were analyzed with one-way repeated measures ANOVAs and subsequent Bonferroni post hoc tests. 

Before performing cluster analysis, all scores were transformed into z-scores. The squared Euclidian distance was used as a similarity measure [30]. Variables used in the cluster analysis were individual results on the two subscales HAMD-21 and Atyp-8 (from the SIGH-SAD/SR), the total score on the fatigue questionnaire (FQ), and the Epworth Sleepiness Scale (ESS). Subgroups were identified with Ward's method, which is a hierarchical agglomerative cluster analysis method that does not require prior knowledge of the number of clusters. It is suitable for small samples, and results are presented graphically in a dendrogram [30]. Differences between clusters at baseline were analyzed with one-way ANOVAs. Validation of clusters on demographic variables was performed with Chi-square tests (nominal data), Fisher's exact tests (when expected values for the number of cases/cells were <5), or Kruskal-Wallis test (ordinal data). Demographic variables were dichotomized before performing the validation analysis. Differential outcomes (after BLT) in the clusters were analyzed with group × time repeated measure ANOVAs (results presented in box plots). A two-tailed *P* value of .05 was used. All analyses were performed in SPSS, version 15.

## 3. Results

### 3.1. Effects of Bright Light Therapy for the Total Group on Measures of Fatigue, Sleepiness, and Health-Related Quality of Life

Mean values on the FQ, the ESS, the SF-36, and VAS-ratings of mood and sleepiness at baseline, posttreatment, and at the one-month followup are presented in Table 1 (*N* = 47). Scores on all measures improved, and results were maintained at the one-month followup.

### 3.2. Identifying and Exploring Subgroups with Cluster Analysis

**Figure 1:**
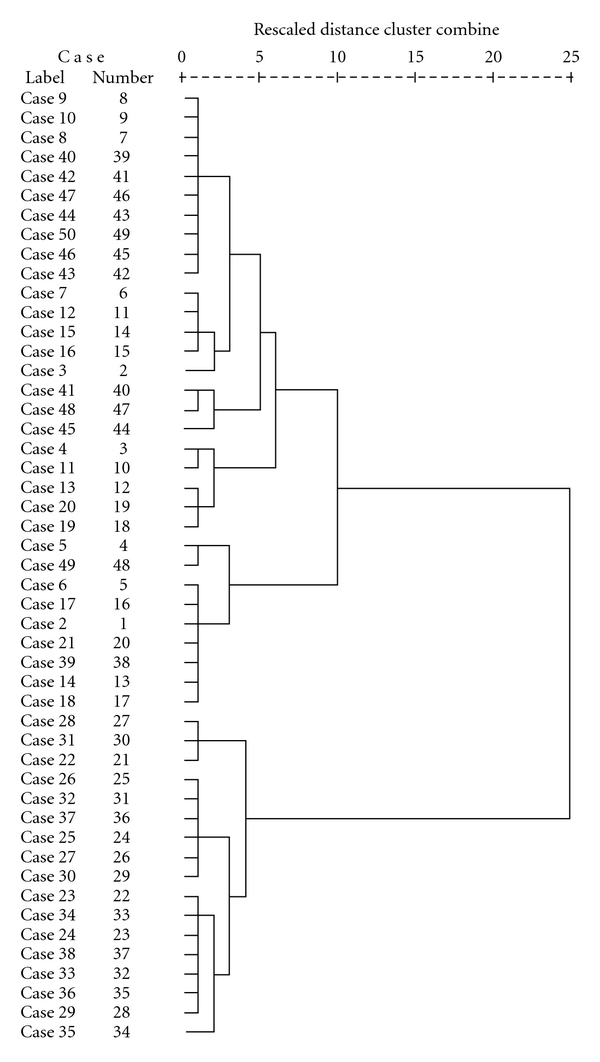
A hierarchical cluster analysis (Ward's method) of subjects with winter SAD and S-SAD (*N* = 49) based on baseline data taken from four measures: the fatigue questionnaire (FQ), the Epworth Sleepiness Scale (ESS), and the two subscales, the HAMD-21 and Atyp-8 (from the depression scale SIGH-SAD/SR).

Results from the cluster analysis are presented in Figure 1. A visual inspection of the dendrogram based on the size of and relative distance between the clusters resulted in the choice of a three-cluster solution for further analysis. Mean values for the clustering variables in each cluster are presented in Table 2. The clusters were characterized primarily on the basis of differential degrees of depression and sleepiness. Even though fatigue scores also differed between the clusters, these scores were high compared to population norms in all three clusters. On the basis of these similarities and differences, the condition winter SAD and S-SAD was labelled “winter fatigue,” and the three subgroups presented according to the level of depression and sleepiness, that is, “mildly depressed, not sleepy,” “mildly depressed, sleepy,” and “depressed, sleepy” (Table 2).

### 3.3. Validation of Clusters on Demographic Variables, Other Measures, and Over Time

There were no statistically significant differences between clusters on the variables age, sex, civil status, education, duration of seasonal symptoms, comorbid disorders, or medication. A difference between the clusters was found for one variable; that is, present occupation (working versus being sick listed or retired (Fischer's exact test, *P* = .047). However, this difference between the clusters was not considered to have any major impact on the interpretation of results since the majority in the sample was in fact employed (*N* = 44/49).

Between-groups ANOVA showed significant differences between the clusters on the SF-36, VAS, and the SPAQ GS-score (Table 3). The “depressed-sleepy” cluster had lower scores than the other two clusters on all scales in the SF-36. Several of the subscale scores were strikingly low for the “depressed-sleepy” cluster. All the three clusters had low scores on the MCS (mental component summary scale) and the VT (vitality) subscale. The PCS (physical component summary score) was lower compared to norms in the “depressed-sleepy” subgroup only. The perception of general health (GH) and bodily pain (BP) differed between the two mildly depressed clusters: the “mildly depressed-not sleepy” and the “mildly depressed-sleepy” clusters (Table 3). To summarize, the mental health scales in the SF-36 were notably low for all clusters, while the physical health scales were low in the “depressed-sleepy” subgroup only. 

Results on the VAS sleepiness scale were consistent with other measures, that is, were lower in the two subgroups labeled “sleepy,” that is, the “mildly depressed-sleepy” and “depressed-sleepy” clusters. Levels of the VAS mood scale did not differ between the clusters. The SPAQ GS-score was higher (worse) in the “depressed-sleepy” cluster compared with the other two clusters.

**Figure 2:**
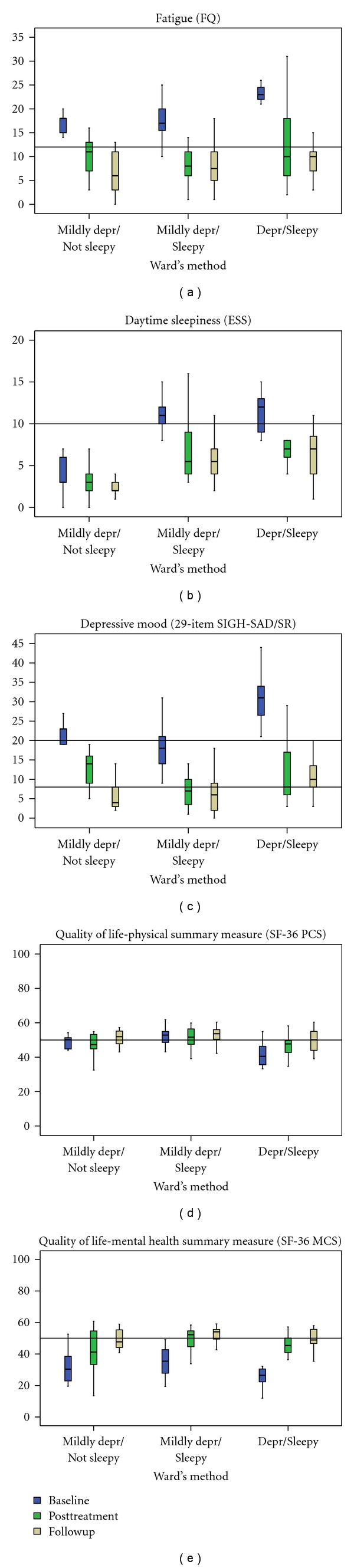
The box plots illustrate results from ANOVA repeated measures analysis of the clusters/subgroups at baseline, posttreatment, and one-month followup. A reference (horizontal) line is given for each measure; the fatigue questionnaire (FQ), a population mean set at 12.2 [25] and the Epworth Sleepiness Scale (ESS), a cutoff for excessive daytime sleepiness set at ≥10 [26]. Two reference lines are given for the 29-item SIGH-SAD/SR depression scale, one representing scores for individuals without depressive symptoms set at ≤8 [31] and the other indicating possible depression set at ≥20 [32]. The reference lines for the SF-36 PCS (physical component summary scale) and MCS (mental component summary scale) represent mean values for the general population set at 50 [27].

When evaluating differential outcome of BLT in the three clusters, there were significant group x time interactions for the ESS, *F*(4,86) = 5.3, *P* ≤ .001 and the 29-item SIGH-SAD/SR total score, *F*(3.4,74.3) = 6.2, *P* < .001. There were no such group x time interactions for the FQ, *F*(3.5,76.8) = 1.9, n.s., the SF-36 PCS, *F*(4,88) = 2.3, n.s., the SF-36 MCS, *F*(4,88) = 1.5, n.s., the VAS mood scale, *F*(3.3,72.4) = 0.3, n.s., or the VAS sleepiness scale, *F*(3.4,75.0) = 0.5, n.s. The median values at baseline, posttreatment, and the one-month followup including some commonly used cutoffs/population means are presented in Figure 2.

## 4. Discussion

A main finding in this study was that fatigue, excessive daytime sleepiness, and health-related quality of life were improved in persons with seasonal affective disorder (SAD) and subsyndromal SAD following treatment in a light room. Results were maintained for at least one month. The results correspond to and strengthen conclusions drawn in the previous randomised controlled study, where the level of depression was reduced after treatment in a light room [18]. 

There are, to our knowledge, only one previous study investigated effects on HRQOL following treatment with BLT in a light room setting and including persons with SAD [33]. Results from that study are consistent with the present results and suggest that patients with SAD have a marked impairment of HRQoL during the winter months and that treatment results in a corresponding marked improvement in both depressed mood and HRQOL. Another study comparing winter versus summer scores indicates that even though scores for HRQOL in patients with SAD were markedly low during the winter, they were significantly improved and within the normal range during the summer [34]. 

Subjective ratings of mental health (the subscale MCS in the SF-36) were much lower in the sample at baseline compared with the Swedish normative data [27]. Results for the MCS in our study (mean value 32) were well below a previously proposed cutoff for possible depression (mean value ≤42) [35]. The degree of impairment corresponds to results from studies in both seasonal and nonseasonal depression [33, 34, 36, 37]. The SF-36 VT (vitality) subscale was remarkably low at baseline in our study (mean value 30.1, SD = 16.8). A corresponding low figure in a Swedish general population sample correlates to low ratings of global health [27]. This is a reminder that there may be substantial subjective functional impairment not only in depression, but in subsyndromal depression as well. Following treatment and at the one-month followup, HRQOL scores were substantially improved and equal to or close to population norms. 

A series of studies performed in other settings than light rooms from a research group in Finland show that bright light exposure was effective in improving depressive mood and HRQOL in healthy subjects with or without seasonal symptoms [38] and that both exercise and bright light alone or in combination was effective in relieving depressive mood and improving HRQOL in healthy employees with S-SAD [39, 40]. Results from a large cross-sectional study (*N* > 6600) suggested that in the population, HRQoL is influenced by seasonal changes and the illumination experienced indoors [41]. Preliminary data suggest that outdoor work during the winter season may have a positive effect on mood [42].

Excessive daytime sleepiness (EDS) is a symptom commonly encountered in clinical practice and may arise from many somatic and psychiatric disorders. It is also related to lifestyle behaviors [43]. It is conceptualized as the drive to fall asleep at inappropriate times and despite attempts to remain awake, and it is a condition which is relieved by good sleep [44]. Patient complaints may be expressed in terms of “tiredness,” “fatigue,” or “lack of energy” rather than a direct reference to impaired alertness or excessive sleepiness [43]. Fatigue, on the other hand, has been defined as an overwhelming sense of tiredness, lack of energy, and feeling of exhaustion [45]. It is a subjective, unpleasant symptom which incorporates total body feelings ranging from tiredness to exhaustion creating an unrelenting overall condition which interferes with the individuals' ability to function to normal capacity [46]. In contrast to EDS, in the short-term perspective, fatigue is not relieved by sleep. In sleep medicine, sleepiness and fatigue are considered two different but related concepts with partial overlap that need separate assessments *琁*[47, 48]. Both EDS and fatigue are common symptoms in major depressive disorders. For example, prevalence figures for fatigue in major depressive disorder range between 73 and 93% [4, 49], but fatigue is not specific for depression; it can be measured independently of depression and is prevalent in a large number of other psychiatric and somatic disorders as well [50]. Excessive daytime sleepiness (EDS) is commonly viewed as a sign of disturbed or inadequate sleep and is associated with different types of sleep disorders (such as obstructive sleep apnea). There is evidence for an association between EDS and moderate-to-severe depression, [51]. There are several questionnaires that rate the severity of EDS; one of the most frequently used is the Epworth Sleepiness Scale (ESS), which was used in the present study [26, 44]. There are at least fifty different questionnaires that rate the severity of fatigue [52], the majority of these being disease specific and not suitable for the present application. 

To our knowledge, this is the first time the fatigue questionnaire (FQ) and the Epworth Sleepiness Scale (ESS) were used in a controlled clinical study of BLT in SAD and S-SAD. Fatigue (FQ) was markedly high at baseline (mean value 19.3) compared with that of the general population (mean value 12.2) [25]. The increase in daytime sleepiness (ESS) was more moderate at baseline (mean value 10.0). A cutoff of ≥10 on the ESS is commonly used to indicate excessive daytime sleepiness which contrasts to that of healthy controls (mean value 5.9) but is not indicative of a high level of daytime sleepiness [26]. The scores on the ESS improved following BLT, which is consistent with another clinical study of BLT in SAD in which measures of excessive daytime sleepiness were used [53]. 

Common features in the subgroups, independent of the level of depressed mood, were a high level of fatigue (the FQ) and low estimates of subjective mental health (the MCS in the SF-36). These results indicate that there are similarly low ratings of mental health among persons with SAD and subsyndromal SAD. Therefore, one may argue that fatigue rather than depressive mood is the common core symptom in SAD and S-SAD. In spite of the differences in baseline severity of depression and sleepiness, all the three subgroups benefited from BLT. At the one-month followup, scores for fatigue (the FQ), excessive daytime sleepiness (the ESS), and health-related quality of life (the SF-36) were within (or close to) the normal range in all three subgroups (Figure 2). Thus, even though clustering revealed distinct subgroups at baseline, there were more similarities between subgroups than were differences over time. Therefore, we suggest the term “winter fatigue” for the combined group of persons with SAD and S-SAD and for the subgroups a label according to the symptoms. More specifically, “simple winter fatigue” (the “Mildly depressed-Not sleepy subgroup), “Winter fatigue with sleepiness” (the mildly depressed-sleepy subgroup), and “Winter depression” (the depressed-sleepy subgroup) are suggested as more convenient labels for the clusters. It is not the first time such a suggestion is made; in 1986, Mueller and Davies proposed that seasonal affective disorder should be classified as a seasonal energy syndrome [54].

Since cluster analysis always produces clusters, it is essential to go through a validation procedure to evaluate whether clusters are useful or meaningful for the clinician. Group differences between the clusters on the clustering variables, on independent variables and over time (group x time interaction), are three of the most recommended validation methods [30] and were all used in the present study. The clusters were validated on cluster variables, demographic variables, and other independent measures at baseline. However, there were similar treatment effects over time. The clusters/subgroups should be regarded as preliminary until validated in further studies of independent samples. The majority of subjects in the present study were considered to have a mild-to-moderate level of depressive mood, and results can therefore not be generalized to more severe seasonal depression. 

The ESS had an unexpectedly low correlation to all the other measures in this study. This result contradicts results from other studies, in which the level of depression was positively associated with the level of sleepiness [55]. A possible explanation may be that, in this sample, some persons were later diagnosed as having comorbid sleep disorders, such as snoring and delayed sleep phase syndrome. It is well-known that a portion of depressed patients may have an undiagnosed comorbid sleep-disordered breathing, mostly obstructive sleep apnea [51, 56, 57], and the same may be true for persons with a seasonal depression. The sample in the present study was recruited from a random sample of the general population and not from a patient population. This procedure made it possible to identify persons with S-SAD for inclusion in the study. However, even though all subjects were clinically diagnosed by an experienced psychiatrist, the risk of missing comorbid disorders may be higher in such a sample compared to a sample from a patient population. The results from the cluster analysis can possibly be interpreted in a similar way since the three empirically derived subgroups were differentiated mainly by two variables; depressive mood (the SIGH-SAD/SR) and excessive daytime sleepiness (the ESS). In the clinic, this may be a reminder that comorbid sleep disorders are common and should be considered when patients report seasonal mood changes. 

In conclusion, fatigue, sleepiness, and health-related quality of life improved in a similar way as measures of depression in persons with SAD and S-SAD following treatment with BLT. Exploring treatment effects in empirically derived clusters showed that all the three subgroups benefited from bright light therapy and that the effects were maintained during the one-month followup. A common feature in the clusters was a high level of fatigue, while levels of depression and sleepiness differed between groups. The results support common features in SAD and S-SAD rather than differences and point to the usefulness of including a broader range of outcome measures in future clinical trials.

##  Conflict of Interests

The authors declare that they have no conflict of interests.

## Figures and Tables

**Table 1:** Mean values (SD) for daytime sleepiness, fatigue, and quality of life in subjects with mild-to-moderate winter depressive mood at baseline, posttreatment, and at the one-month followup (*N* = 47). Treatment refers to bright light therapy in a light room.

Measure	Baseline		Posttreatment		Followup		Within-subjects repeated measures ANOVA	
	Mean	(SD)	Mean	(SD)	Mean	(SD)	*F*(df)^5^	*P* value
Fatigue questionnaire (FQ)^1^	19.3	(4.2)	10.7	(7.7)	9.5	(9.8)	24.7(1.7,79.4)	<.001
Epworth Sleepiness Scale (ESS)^2^	10.0	(4.1)	6.4	(4.0)	5.5	(3.1)	59.1 (2,90)	<.001

VAS^3^								
Mood	49.5	(11.7)	65.5	(15.6)	70.4	(15.1)	57.1 (1.6,75.8)	<.001
Sleepiness	36.1	(14.9)	60.8	(20.0)	68.5	(14.2)	82.8 (1.7,77.9)	<.001

SF-36^4^								
Physical health (PCS)	47.7	(7.4)	49.0	(6.7)	51.1	(6.6)	6.0 (2,92)	=.004
Physical functioning (PF)	83.3	(12.9)	87.3	(11.5)	90.2	(10.9)	11.7 (1.8,80.6)	<.001
Role—physical (RP)	58.5	(33.1)	78.2	(31.1)	90.4	(19.2)	20.0 (2,92)	<.001
Bodily pain (BP)	57.6	(26.8)	71.7	(23.6)	76.6	(23.6)	17.6 (1.8,80.5)	<.001
General health (GH)	63.8	(23.1)	70.9	(19.0)	75.4	(17.0)	14.6 (2,92)	<.001
Mental health (MCS)	31.8	(10.4)	46.2	(11.0)	49.8	(8.9)	66.7 (2,92)	<.001
Vitality (VT)	30.1	(16.8)	61.9	(22.6)	69.1	(17.1)	82.5 (2,92)	<.001
Social functioning (SF)	62.8	(23.4)	80.9	(21.8)	91.5	(14.1)	35.8 (2,92)	<.001
Role—emotional (RE)	43.3	(41.1)	80.1	(29.2)	88.7	(24.4)	43.5 (1.6,72.3)	<.001
Mental health (MH)	56.4	(16.6)	75.1	(18.4)	79.6	(14.5)	38.4 (2,92)	<.001

	N	(%)	N	(%)	N	(%)		
Proportion with ESS total score ≤8^6^	13	(27.7)	38	(80.0)	39	(84.8)		
Proportion with FQ total score ≤12^7^	3	(6.4)	34	(72.3)	39	(83.0)		

^1^High values correspond to high level of fatigue (feeling worse).

^2^High values correspond to high level of sleepiness (feeling worse).

^3^Four uni- or Two bivariate 0–100 visual analogue scales. Low values correspond to high level of depressive mood/sleepiness (feeling worse).

^4^Low values correspond to lower level of functioning (feeling worse).

^5^When Mauchly's test of sphericity was significant, a Greenhouse-Geisser correction of degrees of freedom was used. *P* values refer to differences between baseline and the one-month followup.

^6^Scores <8 are considered to be within the normal range [26].

^7^The score 12 represents approximately the population mean [25].

**Table 2:** Mean values (SD) for the cluster variables in the three clusters at baseline (*N* = 49).

Cluster variables	Winter fatigue				
	Mildly depr/Not Sleepy	Mildly depr/Sleepy	Depressed/Sleepy	Between-groups ANOVA	
	*N* = 9	*N* = 23	*n* = 17	*F*(2,48)	*P* value
HAMD-21 (SD)	12.9 (4.1)	11.4 (5.0)	20.4 (6.2)	14.7	<.001
Atyp-8 (SD)	6.9 (2.8)	6.3 (2.7)	12.3 (2.6)	26.3	<.001
FQ (SD)	16.9 (2.1)	17.7 (4.2)	23.4 (1.8)	18.8	<.001
ESS (SD)	3.8 (2.2)	11.7 (3.1)	11.5 (2.6)	28.7	<.001

**Table 3:** Results for the three clusters on independent measures at baseline, that is, other measures than those used in the formation of clusters at baseline (*N* = 49).

Measure	Winter fatigue							
	Mildly depr-Not Sleepy		Mildly depr-Sleepy		Depressed-Sleepy		Between-groups ANOVA	
	Mean	(SD)	Mean	(SD)	Mean	(SD)	*F*(2,48)	*P* value
SF-36^1^								
Physical health (PCS)	48.8	(4.2)	51.3	(6.3)	40.5	(6.9)	15.2	<.001
Physical functioning (PF)	85.6	(11.8)	87.8	(10.4)	71.8	(15.9)	8.1	≤.001
Role—physical (RP)	72.2	(19.5)	73.9	(27.7)	23.5	(24.2)	21.8	<.001
Bodily pain (BP)	56.7	(29.6)	67.1	(21.3)	42.2	(26.9)	4.9	≤.01
General health (GH)	59.0	(20.4)	76.0	(17.2)	47.6	(21.7)	10.6	<.001
Mental health (MCS)	33.1	(11.9)	35.3	(9.2)	24.7	(8.8)	6.1	=.004
Vitality (VT)	33.3	(17.7)	34.3	(15.5)	19.1	(16.5)	4.7	≤.01
Social functioning (SF)	70.8	(27.9)	67.9	(18.8)	45.6	(24.9)	5.8	=.006
Role—emotional (RE)	48.1	(44.4)	57.9	(40.5)	17.6	(26.7)	5.9	=.005
Mental health (MH)	56.0	(17.8)	63.1	(12.2)	44.0	(18.5)	7.3	=.002

VAS^2^								
Mood (very sad-very happy)	53.3	(16.8)	50.1	(11.2)	43.3	(11.3)	2.4	n.s
Sleepiness (very sleepy-very alert)	45.2	(16.5)	37.6	(12.4)	27.7	(13.8)	5.4	=.008

SPAQ^3^								
Global seasonal score (GS score)	12.5	(2.9)	11.8	(2.2)	15.5	(2.9)	10.1	<.001

^1^The SF-36, high values correspond to feeling better.

^2^Two bivariate 0–100 visual analogue scales. High values correspond to feeling better.

^3^GS score from the Seasonal Pattern Assessment Questionnaire (SPAQ). High values correspond to more seasonal symptoms.
